# Needs of families at‐risk of *APP*_V717I_ and *PSEN1*_A431E_ autosomal dominant Alzheimer's disease in Jalisco, Mexico

**DOI:** 10.1002/alz70858_105181

**Published:** 2025-12-26

**Authors:** Sofía C Arboleya‐García, Angélica Zuno‐Reyes, Isis Medina‐Román, Aranzazú González‐Bravo, Isaac Enrique Berumen‐Ocegueda, Karina Pérez‐Rubio, Ana Karen Preciado‐Baron, Frida Rosales‐Leycegui, Luis E Figuera, John M Ringman, Esmeralda Matute

**Affiliations:** ^1^ Instituto de Neurociencias, CUCBA, Universidad de Guadalajara, Guadalajara, JA, Mexico; ^2^ Centro Universitario de Tonalá, Universidad de Guadalajara, Tonalá, JA, Mexico; ^3^ Instituto Mexicano del Seguro Social, Guadalajara, JA, Mexico; ^4^ University of Southern California, Los Angeles, CA, USA; ^5^ Universidad de Guadalajara, Guadalajara, JA, Mexico

## Abstract

**Background:**

A relationship of trust between researchers and participants promotes their continued involvement in the studies, enhanced by community networking (CN) meetings. Considering the above, our research group conducts regular meetings with families at risk of *APP*_V717I_ and *PSEN1*_A431E_ autosomal dominant Alzheimer's disease (ADAD) in Jalisco, Mexico. As a result of our last CN meetings, we aimed to explore the needs expressed by these families to guide our future work.

**Method:**

Ninety‐one family members, including preclinical, clinical, and non‐consanguineous relatives, attended our three 2024 CN meetings. Site 1 ‐ *PSEN1*_A431E_ (*n* = 33; 20 women), Site 2 ‐ *APP*_V717I_ (*n* = 36; 22 women), and Site 3 ‐ *PSEN1*_A431E_ (*n* = 22; 11 men). At the end of each CN meeting, we collected voluntary, anonymous written responses about their needs through two open questions handed out individually: 1. What recommendations would you make for future meetings? 2. What are your Alzheimer's‐related needs? We coded the responses, analyzed their frequency, and based on this, generated consensual response categories.

**Results:**

We obtained seventy‐seven responses for both questions, fifty‐eight for question 1, and fifty‐nine for question 2. For the first question, nineteen family members did not answer; for the second question, eighteen did not. Then, we classified all of them into four categories: Research group efforts (*n* = 47; e.g., enrollment and workshops), Health Information (*n* = 39; e.g., prevention and treatments), Caregiving (*n* = 26; e.g., care institutions and supplies), Psychosocial Support (*n* = 11; e.g., support groups and genetic counseling). Site 1 was the only one requesting genetic counseling (*n* = 3) and paid care facilities (*n* = 9). Site 3 was the only that requested care supplies, such as nappies and wheelchairs (*n* = 3).

**Conclusions:**

Most of the answers reflected a common interest related to our Research group efforts. However, Health Information, Caregiving, and Psychological support were also highlighted. Each site expressed concerns that must be related to the context and lifestyle (e.g., occupations that complicate caregiving, genetic status, motor difficulties). This study enables us to develop future work tailored to the family's needs.

